# Bis{μ-[(di­phenyl­phosphor­yl)meth­yl](phen­yl)bis­(1*H*-pyrazol-1-yl)boranuido}dilithium

**DOI:** 10.1107/S1600536814011945

**Published:** 2014-05-31

**Authors:** Manuela Müller, Hans-Wolfram Lerner, Michael Bolte

**Affiliations:** a Institut für Anorganische Chemie, J. W. Goethe-Universität Frankfurt, Max-von-Laue-Strasse 7, 60438 Frankfurt/Main, Germany

## Abstract

The title compound, [Li_2_(C_25_H_23_BN_4_OP)_2_], features a centrosymmetric dimeric complex. The four-memberered Li_2_O_2_ ring is exactly planar due to symmetry. The Li atom is four-coordinated by two O atoms and by two N atoms of two different pyrazole rings. The dihedral angle between two pyrazole rings bonded to the same B atom is 45.66 (9)°. The B—N—N—Li—N—N metalla ring adopts a boat conformation. The crystal packing is stabilized by van der Waals inter­actions only.

## Related literature   

For background to scorpionates, see: Trofimenko (1993 ▶, 1999 ▶); Bieller *et al.* (2006 ▶). For related structures, see: Müller *et al.* (2014*a* ▶,*b* ▶).
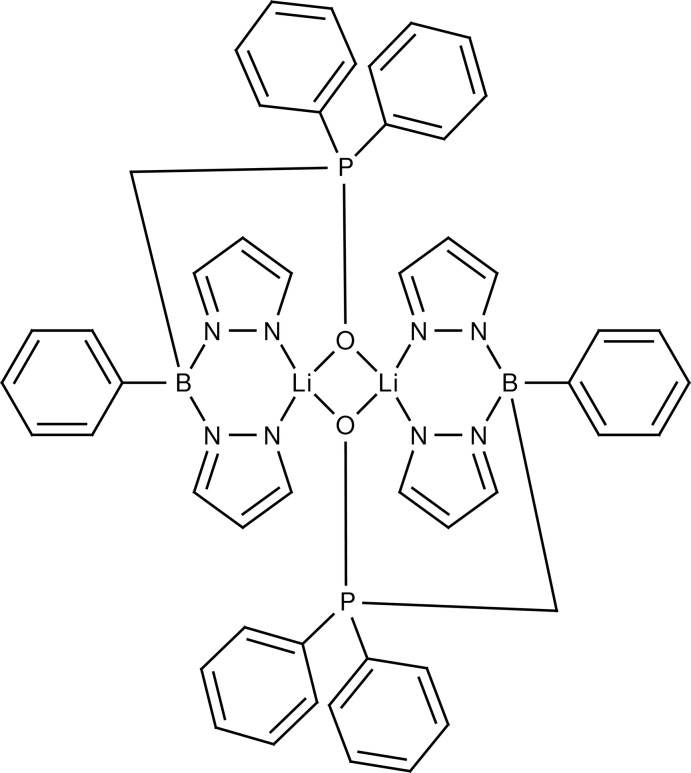


## Experimental   

### 

#### Crystal data  

[Li_2_(C_25_H_23_BN_4_OP)_2_]
*M* *_r_* = 888.39
Monoclinic, 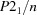

*a* = 10.0585 (6) Å
*b* = 16.2371 (8) Å
*c* = 14.4301 (8) Å
β = 98.854 (5)°
*V* = 2328.7 (2) Å^3^
*Z* = 2
Mo *K*α radiation
μ = 0.14 mm^−1^
*T* = 173 K
0.32 × 0.28 × 0.27 mm

#### Data collection  

Stoe IPDS II two-circle diffractometer
Absorption correction: multi-scan (*MULABS*; Spek, 2009 ▶; Blessing, 1995 ▶) *T*_min_ = 0.956, *T*_max_ = 0.962
36952 measured reflections
4362 independent reflections
3630 reflections with *I* > 2σ(*I*)
*R*_int_ = 0.062

#### Refinement  

*R*[*F* ^2^ > 2σ(*F* ^2^)] = 0.041
*wR*(*F* ^2^) = 0.101
*S* = 1.02
4362 reflections
298 parameters
H-atom parameters constrained
Δρ_max_ = 0.36 e Å^−3^
Δρ_min_ = −0.29 e Å^−3^

### 

Data collection: *X-AREA* (Stoe & Cie, 2001 ▶); cell refinement: *X-AREA*; data reduction: *X-AREA*; program(s) used to solve structure: *SHELXS97* (Sheldrick, 2008 ▶); program(s) used to refine structure: *SHELXL97* (Sheldrick, 2008 ▶); molecular graphics: *XP* in *SHELXTL* (Sheldrick, 2008 ▶); software used to prepare material for publication: *SHELXL97* and *publCIF* (Westrip, 2010 ▶).

## Supplementary Material

Crystal structure: contains datablock(s) I, global. DOI: 10.1107/S1600536814011945/sj5403sup1.cif

Structure factors: contains datablock(s) I. DOI: 10.1107/S1600536814011945/sj5403Isup2.hkl

CCDC reference: 883935

Additional supporting information:  crystallographic information; 3D view; checkCIF report

## Figures and Tables

**Fig. 1.**
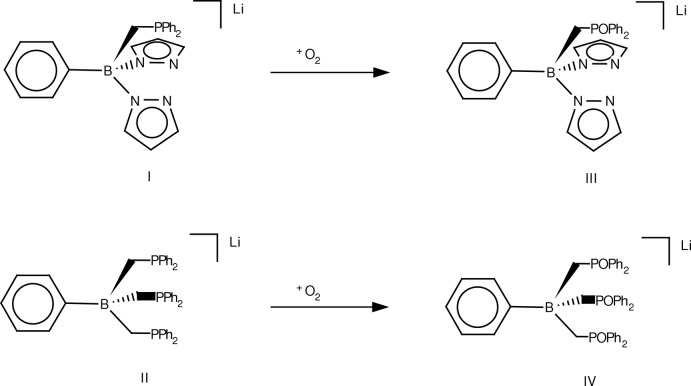
Oxidation of diphenylphosphanylmethyl-substituted heteroscorpionates Li[PhBpzn(CH2PPh2)3-n] (n = 2, 0) with O2.

**Fig. 2.**
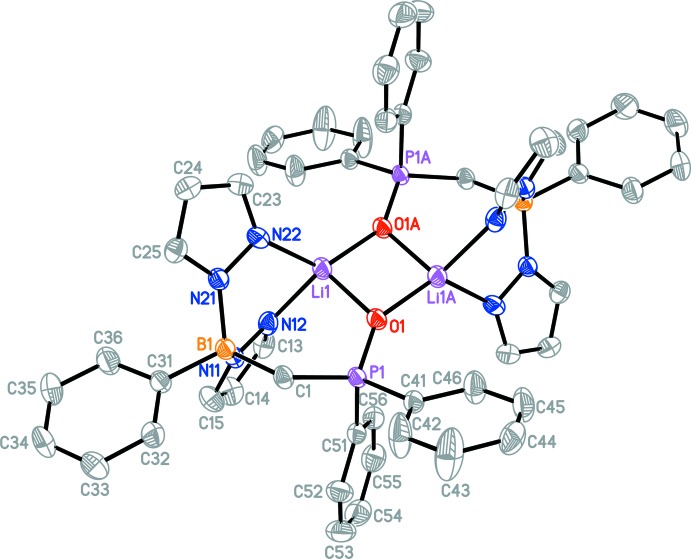
Perspective view of the title compound with displacement ellipsoids drawn at the 50% probability level. Hydrogen atoms omitted for clarity.

**Fig. 3.**
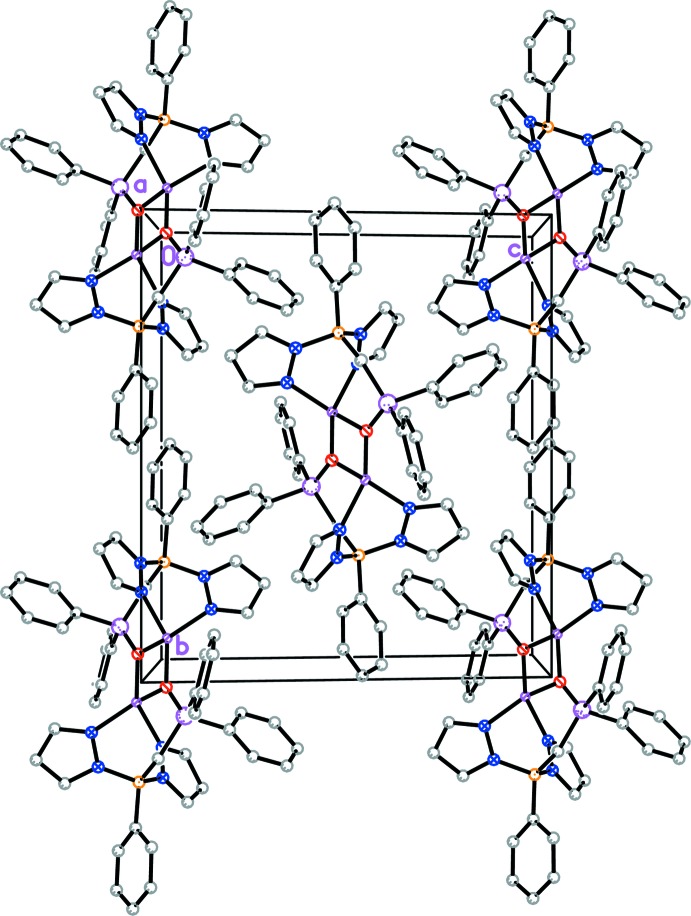
Packing diagram of the title compound with view onto the bc plane. Hydrogen atoms omitted for clarity.

## supplementary crystallographic information

### 1. Comment

One way to alter the donor/acceptor properties of scorpionate ligands over a wider range is to replace the pyrazolyl rings by phosphorus-containing groups (Trofimenko, 1993; Trofimenko, 1999; Bieller *et al.*, 2006). Similar to parent scorpionates these ligands provide a monoanionic, tridentate, face-capping coordination mode, but they differ from parent scorpionates with regard to the softness of their donor sets. Recently we have investigated the syntheses of the hybrid scorpionates Li(tmeda)[PhBpz_2_(CH_2_PPh_2_)] (I) and Li(tmeda)_2_[PhB(CH_2_PPh_2_)_3_] (II) (Müller *et al.*, 2014*a*). The lithium scorpionates I and II, however, are air-sensitive. The heteroscorpionates I and II react with oxygen from the air to give the corresponding oxo-heteroscorpionates as shown in the scheme (Fig. 1). After storing solutions of I and II under ambient conditions (in the presence of oxygen from the air) crystals of the oxo-heteroscorpionates III and IV could be isolated (Müller *et al.*, 2014*b*).

The title compound features a centrosymmetric dimeric complex (Fig. 2). The four-memberered Li_2_O_2_ ring is exactly planar due to the symmetry. Each Li centre is four-coordinated by two oxygen atoms and two nitrogen atoms of two different pyrazol rings. The dihedral angle between two pyrazol rings bonded to the same boron atom is 45.66 (9)°. The B—N—N—Li—N—N ring adopts a boat conformation. The crystal packing is stabilized only by van der Waals interactions (Fig. 3).

### 2. Experimental

Single crystals of the oxo-scorpionate III were grown from a thf solution of I in the presence of air oxygen at r.t.

### 3. Refinement

H atoms were refined using a riding model, with C_aromatic_ —H = 0.95 Å or C_methylene_ —H = 0.99 Å and with *U*_iso_(H) = 1.2*U*_eq_(C).

### Crystal data

**Table:**

[Li_2_(C_25_H_23_BN_4_OP)_2_]	*F*(000) = 928
*M**_r_* = 888.39	*D*_x_ = 1.267 Mg m^−^^3^
Monoclinic, *P*2_1_/*n*	Mo *K*α radiation, λ = 0.71073 Å
Hall symbol: -P 2yn	Cell parameters from 30927 reflections
*a* = 10.0585 (6) Å	θ = 3.5–25.8°
*b* = 16.2371 (8) Å	µ = 0.14 mm^−^^1^
*c* = 14.4301 (8) Å	*T* = 173 K
β = 98.854 (5)°	Block, colourless
*V* = 2328.7 (2) Å^3^	0.32 × 0.28 × 0.27 mm
*Z* = 2	

### Data collection

**Table:**

Stoe IPDS II two-circle diffractometer	4362 independent reflections
Radiation source: fine-focus sealed tube	3630 reflections with *I* > 2σ(*I*)
Graphite monochromator	*R*_int_ = 0.062
ω scans	θ_max_ = 25.6°, θ_min_ = 3.5°
Absorption correction: multi-scan (*MULABS*; Spek, 2009; Blessing, 1995)	*h* = −12→12
*T*_min_ = 0.956, *T*_max_ = 0.962	*k* = −19→19
36952 measured reflections	*l* = −17→16

### Refinement

**Table:**

Refinement on *F*^2^	Primary atom site location: structure-invariant direct methods
Least-squares matrix: full	Secondary atom site location: difference Fourier map
*R*[*F*^2^ > 2σ(*F*^2^)] = 0.041	Hydrogen site location: inferred from neighbouring sites
*wR*(*F*^2^) = 0.101	H-atom parameters constrained
*S* = 1.02	*w* = 1/[σ^2^(*F*_o_^2^) + (0.0511*P*)^2^ + 0.9981*P*] where *P* = (*F*_o_^2^ + 2*F*_c_^2^)/3
4362 reflections	(Δ/σ)_max_ = 0.001
298 parameters	Δρ_max_ = 0.36 e Å^−^^3^
0 restraints	Δρ_min_ = −0.29 e Å^−^^3^

### Special details

**Table:**

Geometry. All e.s.d.'s (except the e.s.d. in the dihedral angle between two l.s. planes) are estimated using the full covariance matrix. The cell e.s.d.'s are taken into account individually in the estimation of e.s.d.'s in distances, angles and torsion angles; correlations between e.s.d.'s in cell parameters are only used when they are defined by crystal symmetry. An approximate (isotropic) treatment of cell e.s.d.'s is used for estimating e.s.d.'s involving l.s. planes.
Refinement. Refinement of *F*^2^ against ALL reflections. The weighted *R*-factor *wR* and goodness of fit *S* are based on *F*^2^, conventional *R*-factors *R* are based on *F*, with *F* set to zero for negative *F*^2^. The threshold expression of *F*^2^ > σ(*F*^2^) is used only for calculating *R*-factors(gt) *etc*. and is not relevant to the choice of reflections for refinement. *R*-factors based on *F*^2^ are statistically about twice as large as those based on *F*, and *R*- factors based on ALL data will be even larger.

### Fractional atomic coordinates and isotropic or equivalent isotropic displacement parameters (Å^2^)

**Table:**

	*x*	*y*	*z*	*U*_iso_*/*U*_eq_	
P1	0.77171 (4)	0.58856 (2)	0.40685 (3)	0.02091 (12)	
O1	0.87612 (11)	0.53113 (7)	0.45806 (8)	0.0246 (3)	
Li1	1.0357 (3)	0.57250 (17)	0.5403 (2)	0.0276 (6)	
B1	0.85890 (18)	0.73774 (11)	0.52436 (13)	0.0222 (4)	
C1	0.73591 (16)	0.67798 (10)	0.47084 (12)	0.0235 (4)	
H1A	0.6761	0.7135	0.4268	0.028*	
H1B	0.6827	0.6595	0.5193	0.028*	
N11	0.98554 (14)	0.73847 (8)	0.47140 (10)	0.0237 (3)	
N12	1.08107 (14)	0.67759 (9)	0.47811 (11)	0.0271 (3)	
C13	1.16767 (18)	0.69986 (12)	0.42279 (14)	0.0335 (4)	
H13	1.2452	0.6689	0.4146	0.040*	
C14	1.1313 (2)	0.77457 (13)	0.37775 (16)	0.0398 (5)	
H14	1.1764	0.8035	0.3346	0.048*	
C15	1.0150 (2)	0.79640 (11)	0.41052 (14)	0.0347 (4)	
H15	0.9635	0.8446	0.3931	0.042*	
N21	0.90523 (14)	0.70587 (8)	0.62812 (10)	0.0236 (3)	
N22	0.97663 (15)	0.63450 (9)	0.64916 (11)	0.0297 (3)	
C23	0.99077 (19)	0.62600 (12)	0.74154 (13)	0.0332 (4)	
H23	1.0377	0.5819	0.7754	0.040*	
C24	0.92834 (18)	0.68974 (11)	0.78261 (13)	0.0305 (4)	
H24	0.9237	0.6977	0.8473	0.037*	
C25	0.87472 (18)	0.73874 (11)	0.70831 (13)	0.0276 (4)	
H25	0.8244	0.7877	0.7127	0.033*	
C31	0.81327 (17)	0.83390 (10)	0.53039 (12)	0.0257 (4)	
C32	0.6922 (2)	0.86585 (12)	0.48509 (14)	0.0359 (4)	
H32	0.6274	0.8293	0.4526	0.043*	
C33	0.6627 (3)	0.95001 (13)	0.48588 (17)	0.0510 (6)	
H33	0.5797	0.9697	0.4530	0.061*	
C34	0.7532 (3)	1.00449 (13)	0.53392 (17)	0.0510 (6)	
H34	0.7330	1.0616	0.5348	0.061*	
C35	0.8735 (2)	0.97487 (12)	0.58076 (17)	0.0473 (6)	
H35	0.9364	1.0116	0.6149	0.057*	
C36	0.9030 (2)	0.89140 (11)	0.57815 (15)	0.0387 (5)	
H36	0.9873	0.8725	0.6099	0.046*	
C41	0.61642 (17)	0.53155 (10)	0.37391 (12)	0.0244 (4)	
C42	0.4899 (2)	0.56546 (14)	0.3715 (2)	0.0575 (7)	
H42	0.4816	0.6219	0.3868	0.069*	
C43	0.3744 (2)	0.51809 (16)	0.3469 (2)	0.0693 (9)	
H43	0.2884	0.5425	0.3456	0.083*	
C44	0.3841 (2)	0.43647 (13)	0.32474 (15)	0.0404 (5)	
H44	0.3052	0.4041	0.3089	0.048*	
C45	0.5090 (2)	0.40206 (12)	0.32563 (18)	0.0498 (6)	
H45	0.5163	0.3457	0.3093	0.060*	
C46	0.6247 (2)	0.44880 (12)	0.35018 (17)	0.0444 (5)	
H46	0.7104	0.4240	0.3508	0.053*	
C51	0.82258 (17)	0.62314 (10)	0.29840 (12)	0.0246 (4)	
C52	0.7426 (2)	0.67760 (12)	0.23831 (14)	0.0352 (4)	
H52	0.6607	0.6977	0.2549	0.042*	
C53	0.7826 (2)	0.70227 (14)	0.15467 (15)	0.0446 (5)	
H53	0.7280	0.7391	0.1141	0.054*	
C54	0.9024 (2)	0.67316 (13)	0.13017 (15)	0.0436 (5)	
H54	0.9294	0.6901	0.0728	0.052*	
C55	0.9827 (2)	0.61951 (13)	0.18918 (15)	0.0397 (5)	
H55	1.0647	0.5999	0.1724	0.048*	
C56	0.94305 (18)	0.59439 (11)	0.27293 (13)	0.0301 (4)	
H56	0.9981	0.5575	0.3131	0.036*	

### Atomic displacement parameters (Å^2^)

**Table:**

	*U*^11^	*U*^22^	*U*^33^	*U*^12^	*U*^13^	*U*^23^
P1	0.0181 (2)	0.0160 (2)	0.0273 (2)	0.00044 (15)	−0.00081 (16)	0.00026 (16)
O1	0.0228 (6)	0.0185 (5)	0.0307 (7)	0.0025 (4)	−0.0022 (5)	0.0004 (5)
Li1	0.0284 (15)	0.0197 (13)	0.0321 (16)	0.0048 (11)	−0.0031 (12)	−0.0027 (11)
B1	0.0218 (9)	0.0194 (9)	0.0250 (10)	0.0012 (7)	0.0020 (8)	−0.0002 (7)
C1	0.0203 (8)	0.0191 (8)	0.0308 (9)	0.0017 (6)	0.0030 (7)	−0.0011 (7)
N11	0.0224 (7)	0.0198 (7)	0.0282 (8)	0.0001 (5)	0.0021 (6)	0.0001 (6)
N12	0.0235 (7)	0.0248 (7)	0.0321 (8)	0.0030 (6)	0.0015 (6)	−0.0069 (6)
C13	0.0251 (9)	0.0348 (10)	0.0414 (11)	−0.0014 (8)	0.0078 (8)	−0.0115 (8)
C14	0.0363 (11)	0.0416 (11)	0.0449 (12)	−0.0064 (9)	0.0169 (9)	0.0035 (9)
C15	0.0362 (10)	0.0277 (9)	0.0423 (11)	−0.0002 (8)	0.0125 (9)	0.0075 (8)
N21	0.0239 (7)	0.0200 (7)	0.0265 (8)	0.0011 (5)	0.0023 (6)	−0.0001 (6)
N22	0.0365 (8)	0.0223 (7)	0.0290 (8)	0.0081 (6)	0.0008 (7)	0.0028 (6)
C23	0.0372 (10)	0.0314 (10)	0.0293 (10)	0.0035 (8)	−0.0009 (8)	0.0044 (8)
C24	0.0294 (9)	0.0380 (10)	0.0236 (9)	−0.0015 (8)	0.0025 (7)	−0.0010 (8)
C25	0.0261 (9)	0.0295 (9)	0.0275 (9)	0.0002 (7)	0.0047 (7)	−0.0048 (7)
C31	0.0304 (9)	0.0216 (8)	0.0253 (9)	0.0017 (7)	0.0052 (7)	0.0012 (7)
C32	0.0388 (10)	0.0288 (9)	0.0376 (11)	0.0076 (8)	−0.0019 (8)	−0.0060 (8)
C33	0.0579 (14)	0.0380 (12)	0.0524 (14)	0.0236 (10)	−0.0065 (11)	−0.0029 (10)
C34	0.0745 (16)	0.0227 (10)	0.0544 (14)	0.0133 (10)	0.0056 (12)	−0.0018 (9)
C35	0.0625 (14)	0.0230 (10)	0.0546 (14)	−0.0049 (9)	0.0033 (11)	−0.0054 (9)
C36	0.0394 (11)	0.0254 (9)	0.0488 (13)	0.0002 (8)	−0.0011 (9)	−0.0010 (8)
C41	0.0228 (8)	0.0203 (8)	0.0287 (9)	−0.0021 (6)	−0.0007 (7)	−0.0005 (7)
C42	0.0247 (10)	0.0355 (11)	0.110 (2)	−0.0012 (9)	0.0024 (12)	−0.0304 (13)
C43	0.0243 (11)	0.0593 (15)	0.124 (3)	−0.0076 (10)	0.0106 (13)	−0.0438 (16)
C44	0.0329 (10)	0.0429 (11)	0.0444 (12)	−0.0181 (9)	0.0028 (9)	−0.0072 (9)
C45	0.0459 (12)	0.0246 (10)	0.0728 (16)	−0.0062 (9)	−0.0100 (11)	−0.0081 (10)
C46	0.0296 (10)	0.0272 (10)	0.0711 (16)	0.0011 (8)	−0.0087 (10)	−0.0109 (10)
C51	0.0250 (8)	0.0201 (8)	0.0272 (9)	−0.0024 (7)	−0.0006 (7)	−0.0015 (7)
C52	0.0329 (10)	0.0341 (10)	0.0367 (11)	0.0035 (8)	−0.0008 (8)	0.0066 (8)
C53	0.0488 (12)	0.0449 (12)	0.0364 (12)	−0.0021 (10)	−0.0052 (10)	0.0125 (9)
C54	0.0553 (13)	0.0462 (12)	0.0298 (11)	−0.0132 (10)	0.0084 (9)	0.0016 (9)
C55	0.0413 (11)	0.0419 (11)	0.0377 (11)	−0.0051 (9)	0.0122 (9)	−0.0071 (9)
C56	0.0314 (9)	0.0270 (9)	0.0314 (10)	−0.0001 (7)	0.0033 (8)	−0.0027 (7)

### Geometric parameters (Å, º)

**Table:**

P1—O1	1.5099 (11)	C31—C32	1.391 (3)
P1—C1	1.7868 (16)	C31—C36	1.404 (3)
P1—C51	1.8092 (18)	C32—C33	1.399 (3)
P1—C41	1.8150 (17)	C32—H32	0.9500
P1—Li1	3.042 (3)	C33—C34	1.377 (3)
O1—Li1^i^	1.901 (3)	C33—H33	0.9500
O1—Li1	1.962 (3)	C34—C35	1.379 (3)
Li1—O1^i^	1.901 (3)	C34—H34	0.9500
Li1—N12	2.013 (3)	C35—C36	1.389 (3)
Li1—N22	2.030 (3)	C35—H35	0.9500
Li1—Li1^i^	2.675 (5)	C36—H36	0.9500
B1—N11	1.583 (2)	C41—C42	1.382 (3)
B1—N21	1.585 (2)	C41—C46	1.392 (3)
B1—C31	1.633 (2)	C42—C43	1.393 (3)
B1—C1	1.666 (2)	C42—H42	0.9500
C1—H1A	0.9900	C43—C44	1.370 (3)
C1—H1B	0.9900	C43—H43	0.9500
N11—C15	1.351 (2)	C44—C45	1.373 (3)
N11—N12	1.3716 (19)	C44—H44	0.9500
N12—C13	1.319 (2)	C45—C46	1.389 (3)
C13—C14	1.398 (3)	C45—H45	0.9500
C13—H13	0.9500	C46—H46	0.9500
C14—C15	1.374 (3)	C51—C56	1.399 (2)
C14—H14	0.9500	C51—C52	1.402 (2)
C15—H15	0.9500	C52—C53	1.389 (3)
N21—C25	1.352 (2)	C52—H52	0.9500
N21—N22	1.3725 (19)	C53—C54	1.390 (3)
N22—C23	1.326 (2)	C53—H53	0.9500
C23—C24	1.390 (3)	C54—C55	1.387 (3)
C23—H23	0.9500	C54—H54	0.9500
C24—C25	1.377 (3)	C55—C56	1.391 (3)
C24—H24	0.9500	C55—H55	0.9500
C25—H25	0.9500	C56—H56	0.9500
			
O1—P1—C1	115.43 (7)	C24—C23—H23	124.3
O1—P1—C51	110.30 (7)	C25—C24—C23	104.29 (16)
C1—P1—C51	107.53 (8)	C25—C24—H24	127.9
O1—P1—C41	108.59 (7)	C23—C24—H24	127.9
C1—P1—C41	108.43 (8)	N21—C25—C24	108.97 (16)
C51—P1—C41	106.16 (8)	N21—C25—H25	125.5
C1—P1—Li1	88.17 (8)	C24—C25—H25	125.5
C51—P1—Li1	103.82 (8)	C32—C31—C36	115.67 (16)
C41—P1—Li1	138.96 (8)	C32—C31—B1	124.36 (16)
P1—O1—Li1^i^	147.24 (12)	C36—C31—B1	119.81 (15)
P1—O1—Li1	121.82 (10)	C31—C32—C33	122.12 (19)
Li1^i^—O1—Li1	87.65 (13)	C31—C32—H32	118.9
O1^i^—Li1—O1	92.35 (13)	C33—C32—H32	118.9
O1^i^—Li1—N12	128.20 (17)	C34—C33—C32	120.5 (2)
O1—Li1—N12	103.93 (14)	C34—C33—H33	119.8
O1^i^—Li1—N22	128.42 (16)	C32—C33—H33	119.8
O1—Li1—N22	109.17 (15)	C33—C34—C35	119.00 (19)
N12—Li1—N22	92.19 (12)	C33—C34—H34	120.5
O1^i^—Li1—Li1^i^	47.13 (9)	C35—C34—H34	120.5
O1—Li1—Li1^i^	45.22 (9)	C34—C35—C36	120.1 (2)
N12—Li1—Li1^i^	128.0 (2)	C34—C35—H35	119.9
N22—Li1—Li1^i^	133.0 (2)	C36—C35—H35	119.9
O1^i^—Li1—P1	116.35 (12)	C35—C36—C31	122.56 (19)
O1—Li1—P1	24.94 (5)	C35—C36—H36	118.7
N12—Li1—P1	83.14 (10)	C31—C36—H36	118.7
N22—Li1—P1	96.69 (11)	C42—C41—C46	117.88 (17)
Li1^i^—Li1—P1	69.52 (11)	C42—C41—P1	123.73 (14)
N11—B1—N21	108.48 (13)	C46—C41—P1	118.38 (14)
N11—B1—C31	105.79 (13)	C41—C42—C43	121.00 (19)
N21—B1—C31	107.82 (13)	C41—C42—H42	119.5
N11—B1—C1	112.07 (13)	C43—C42—H42	119.5
N21—B1—C1	109.71 (13)	C44—C43—C42	120.5 (2)
C31—B1—C1	112.75 (13)	C44—C43—H43	119.8
B1—C1—P1	121.24 (11)	C42—C43—H43	119.8
B1—C1—H1A	107.0	C43—C44—C45	119.26 (18)
P1—C1—H1A	107.0	C43—C44—H44	120.4
B1—C1—H1B	107.0	C45—C44—H44	120.4
P1—C1—H1B	107.0	C44—C45—C46	120.63 (19)
H1A—C1—H1B	106.8	C44—C45—H45	119.7
C15—N11—N12	109.06 (14)	C46—C45—H45	119.7
C15—N11—B1	126.33 (14)	C45—C46—C41	120.73 (19)
N12—N11—B1	124.57 (13)	C45—C46—H46	119.6
C13—N12—N11	106.28 (15)	C41—C46—H46	119.6
C13—N12—Li1	135.92 (15)	C56—C51—C52	119.06 (17)
N11—N12—Li1	115.97 (13)	C56—C51—P1	119.69 (13)
N12—C13—C14	111.66 (16)	C52—C51—P1	121.25 (14)
N12—C13—H13	124.2	C53—C52—C51	120.26 (19)
C14—C13—H13	124.2	C53—C52—H52	119.9
C15—C14—C13	103.87 (17)	C51—C52—H52	119.9
C15—C14—H14	128.1	C52—C53—C54	120.1 (2)
C13—C14—H14	128.1	C52—C53—H53	120.0
N11—C15—C14	109.13 (17)	C54—C53—H53	120.0
N11—C15—H15	125.4	C55—C54—C53	120.2 (2)
C14—C15—H15	125.4	C55—C54—H54	119.9
C25—N21—N22	108.91 (14)	C53—C54—H54	119.9
C25—N21—B1	127.38 (14)	C54—C55—C56	120.00 (19)
N22—N21—B1	123.54 (13)	C54—C55—H55	120.0
C23—N22—N21	106.37 (14)	C56—C55—H55	120.0
C23—N22—Li1	136.66 (15)	C55—C56—C51	120.39 (18)
N21—N22—Li1	116.94 (13)	C55—C56—H56	119.8
N22—C23—C24	111.45 (16)	C51—C56—H56	119.8
N22—C23—H23	124.3		
			
C1—P1—O1—Li1^i^	171.1 (2)	N11—B1—N21—N22	−51.28 (19)
C51—P1—O1—Li1^i^	−66.8 (2)	C31—B1—N21—N22	−165.41 (14)
C41—P1—O1—Li1^i^	49.2 (2)	C1—B1—N21—N22	71.45 (18)
Li1—P1—O1—Li1^i^	−151.0 (2)	C25—N21—N22—C23	−1.26 (19)
C1—P1—O1—Li1	−37.84 (15)	B1—N21—N22—C23	−176.84 (15)
C51—P1—O1—Li1	84.26 (14)	C25—N21—N22—Li1	−179.89 (14)
C41—P1—O1—Li1	−159.79 (13)	B1—N21—N22—Li1	4.5 (2)
P1—O1—Li1—O1^i^	−164.79 (12)	O1^i^—Li1—N22—C23	6.8 (3)
Li1^i^—O1—Li1—O1^i^	0.0	O1—Li1—N22—C23	116.1 (2)
P1—O1—Li1—N12	−34.41 (18)	N12—Li1—N22—C23	−138.3 (2)
Li1^i^—O1—Li1—N12	130.4 (2)	Li1^i^—Li1—N22—C23	70.2 (3)
P1—O1—Li1—N22	62.93 (18)	P1—Li1—N22—C23	138.3 (2)
Li1^i^—O1—Li1—N22	−132.3 (2)	O1^i^—Li1—N22—N21	−175.15 (17)
P1—O1—Li1—Li1^i^	−164.79 (12)	O1—Li1—N22—N21	−65.82 (19)
Li1^i^—O1—Li1—P1	164.79 (12)	N12—Li1—N22—N21	39.75 (17)
O1—P1—Li1—O1^i^	17.01 (14)	Li1^i^—Li1—N22—N21	−111.7 (2)
C1—P1—Li1—O1^i^	163.34 (15)	P1—Li1—N22—N21	−43.60 (16)
C51—P1—Li1—O1^i^	−89.05 (15)	N21—N22—C23—C24	0.9 (2)
C41—P1—Li1—O1^i^	46.9 (2)	Li1—N22—C23—C24	179.15 (18)
C1—P1—Li1—O1	146.34 (14)	N22—C23—C24—C25	−0.2 (2)
C51—P1—Li1—O1	−106.06 (13)	N22—N21—C25—C24	1.13 (19)
C41—P1—Li1—O1	29.91 (19)	B1—N21—C25—C24	176.50 (15)
O1—P1—Li1—N12	146.46 (19)	C23—C24—C25—N21	−0.6 (2)
C1—P1—Li1—N12	−67.20 (10)	N11—B1—C31—C32	114.39 (19)
C51—P1—Li1—N12	40.40 (11)	N21—B1—C31—C32	−129.69 (18)
C41—P1—Li1—N12	176.37 (10)	C1—B1—C31—C32	−8.4 (2)
O1—P1—Li1—N22	−122.13 (19)	N11—B1—C31—C36	−60.8 (2)
C1—P1—Li1—N22	24.21 (11)	N21—B1—C31—C36	55.1 (2)
C51—P1—Li1—N22	131.82 (11)	C1—B1—C31—C36	176.41 (17)
C41—P1—Li1—N22	−92.22 (14)	C36—C31—C32—C33	1.0 (3)
O1—P1—Li1—Li1^i^	11.46 (9)	B1—C31—C32—C33	−174.4 (2)
C1—P1—Li1—Li1^i^	157.80 (14)	C31—C32—C33—C34	−1.3 (4)
C51—P1—Li1—Li1^i^	−94.59 (13)	C32—C33—C34—C35	0.4 (4)
C41—P1—Li1—Li1^i^	41.4 (2)	C33—C34—C35—C36	0.8 (4)
N11—B1—C1—P1	30.97 (19)	C34—C35—C36—C31	−1.2 (4)
N21—B1—C1—P1	−89.61 (16)	C32—C31—C36—C35	0.3 (3)
C31—B1—C1—P1	150.21 (13)	B1—C31—C36—C35	175.9 (2)
O1—P1—C1—B1	49.57 (16)	O1—P1—C41—C42	145.5 (2)
C51—P1—C1—B1	−74.01 (15)	C1—P1—C41—C42	19.4 (2)
C41—P1—C1—B1	171.61 (13)	C51—P1—C41—C42	−95.9 (2)
Li1—P1—C1—B1	29.91 (14)	Li1—P1—C41—C42	128.8 (2)
N21—B1—N11—C15	−140.79 (17)	O1—P1—C41—C46	−34.10 (19)
C31—B1—N11—C15	−25.3 (2)	C1—P1—C41—C46	−160.22 (16)
C1—B1—N11—C15	97.93 (19)	C51—P1—C41—C46	84.50 (18)
N21—B1—N11—N12	41.7 (2)	Li1—P1—C41—C46	−50.9 (2)
C31—B1—N11—N12	157.17 (14)	C46—C41—C42—C43	0.6 (4)
C1—B1—N11—N12	−79.57 (19)	P1—C41—C42—C43	−179.0 (2)
C15—N11—N12—C13	0.96 (19)	C41—C42—C43—C44	0.1 (5)
B1—N11—N12—C13	178.83 (15)	C42—C43—C44—C45	−0.9 (4)
C15—N11—N12—Li1	−166.12 (15)	C43—C44—C45—C46	1.0 (4)
B1—N11—N12—Li1	11.8 (2)	C44—C45—C46—C41	−0.3 (4)
O1^i^—Li1—N12—C13	5.1 (3)	C42—C41—C46—C45	−0.5 (3)
O1—Li1—N12—C13	−99.3 (2)	P1—C41—C46—C45	179.16 (19)
N22—Li1—N12—C13	150.30 (18)	O1—P1—C51—C56	1.27 (16)
Li1^i^—Li1—N12—C13	−56.0 (3)	C1—P1—C51—C56	127.92 (14)
P1—Li1—N12—C13	−113.22 (19)	C41—P1—C51—C56	−116.19 (14)
O1^i^—Li1—N12—N11	167.11 (16)	Li1—P1—C51—C56	35.44 (15)
O1—Li1—N12—N11	62.69 (18)	O1—P1—C51—C52	−179.60 (14)
N22—Li1—N12—N11	−47.68 (16)	C1—P1—C51—C52	−52.95 (16)
Li1^i^—Li1—N12—N11	106.0 (2)	C41—P1—C51—C52	62.94 (16)
P1—Li1—N12—N11	48.80 (13)	Li1—P1—C51—C52	−145.43 (15)
N11—N12—C13—C14	−0.7 (2)	C56—C51—C52—C53	0.2 (3)
Li1—N12—C13—C14	162.48 (19)	P1—C51—C52—C53	−178.95 (15)
N12—C13—C14—C15	0.2 (2)	C51—C52—C53—C54	−0.1 (3)
N12—N11—C15—C14	−0.8 (2)	C52—C53—C54—C55	−0.1 (3)
B1—N11—C15—C14	−178.67 (16)	C53—C54—C55—C56	0.3 (3)
C13—C14—C15—N11	0.4 (2)	C54—C55—C56—C51	−0.2 (3)
N11—B1—N21—C25	133.99 (16)	C52—C51—C56—C55	0.0 (3)
C31—B1—N21—C25	19.9 (2)	P1—C51—C56—C55	179.12 (14)
C1—B1—N21—C25	−103.29 (18)		

Symmetry code: (i) −*x*+2, −*y*+1, −*z*+1.
